# Effect of Corneal Incision Enlargement on Surgically Induced Astigmatism in Biaxial Microincision Cataract Surgery

**DOI:** 10.4274/tjo.52386

**Published:** 2016-06-06

**Authors:** Mehmet Tetikoğlu, Celal Yeter, Fırat Helvacıoğlu, Serdar Aktaş, Hacı Murat Sağdık, Fatih Özcura

**Affiliations:** 1 Dumlupınar University Faculty of Medicine, Department of Ophthalmology, Kütahya, Turkey; 2 Başakşehir State Hospital, Ophthalmology Clinic, İstanbul, Turkey; 3 Maltepe University Faculty of Medicine, Department of Ophthalmology, İstanbul, Turkey

**Keywords:** Astigmatism, biaxial microincision cataract surgery, phacoemulsification

## Abstract

**Objectives:**

To evaluate surgically induced astigmatism (SIA) in biaxial microincision cataract surgery with enlargement of one corneal incision during intraocular lens implantation (IOL).

**Materials and Methods:**

Data from 683 eyes with cataract that underwent biaxial microincision cataract surgery and IOL were retrospectively analyzed. The operated eyes were divided into 4 groups defined by final corneal incision length after IOL implantation. There were 83 eyes with 1.6 mm corneal incisions (group 1) and 200 eyes in each of the 2, 2.4, and 2.8 mm corneal incision groups (groups 2, 3 and 4, respectively). SIA was assessed using preoperative and postoperative keratometric values at one month.

**Results:**

The mean magnitude of SIA was 0.83±0.4 D in group 1, 0.93±0.5 D in group 2, 1.03±0.6 D in group 3 and 1.04±0.7 D in group 4. The SIA showed statistically significant differences between the four groups (p=0.05). Pairwise group comparisons revealed significant differences between groups 1 and 3 and groups 1 and 4 (p=0.005).

**Conclusion:**

Biaxial microincision cataract surgery with an incision size of 1.6 mm resulted in the least SIA. Enlargement of the corneal incision beyond 2.0 mm during IOL implantation led to significant increases in SIA. We believe that with the development and dissemination of IOLs which can be inserted through small corneal incisions, biaxial microincision cataract surgery will be the best choice to prevent SIA and increase visual acuity.

## INTRODUCTION

Modern cataract surgery and improved lens technology have allowed emulsification of the nucleus by phacoemulsification and implantation of intraocular lenses (IOLs) through smaller incisions. Creating smaller incisions minimizes damage to tissues and reduces postoperative pain and inflammation, providing rapid and stable visual rehabilitation. It also minimizes surgically induced astigmatism (SIA), one of the main factors influencing vision quality after cataract surgery.^1,2,3,4,5^ Microincision cataract surgery (MICS) can be performed with either micro-coaxial or biaxial phacoemulsification. With the microaxial procedure, the size of the corneal incision is reduced from 3.2 mm to 2.2 mm, while in biaxial phacoemulsification (B-MICS) the cornea incision is between 0.9 and 1.5 mm.^6,7,8^

Although B-MICS was first described by Shearing et al.^9^ in 1985, it did not begin to gain acceptance among surgeons until much later due to the development of microincision techniques for phacoemulsification. The use of an unsleeved phacoemulsification tip in B-MICS separates irrigation and aspiration. The procedure is performed by making two 1.2-1.5 mm corneal incisions, one for the unsleeved phaco tip, the other for an irrigating chopper.^10,11,12^

One of the major problems in B-MICS is IOL implantation, mainly because it is difficult to insert the lens through the smaller incisions. After surgery with 1.4 mm incisions, there are three approaches to IOL implantation: one of the two existing corneal incisions can be enlarged, a third incision can be created, or the IOL can be implanted without enlarging the microincisions.^13,14,15,16,17^

The aim of this study was to investigate how widening a corneal incision during IOL implantation in B-MICS affects astigmatism.

## MATERIALS AND METHODS

Data from 683 eyes with cataract that underwent B-MICS in Başakşehir State Hospital and the Dumlupınar University Faculty of Medicine, Department of Ophthalmology between January 2011 and April 2014 were analyzed retrospectively. Patients with any preoperative corneal pathologies or intraoperative complications were excluded from the study.

Nuclear hardness was evaluated using the Lens Opacities Classification System II. To investigate the correlation between nuclear hardness and SIA, nuclear hardness grades I-II were evaluated as one group and grades III-IV as another group. Peribulbar anesthesia consisting of 4 ml of 0.0125 mg/ml epinephrine and 2 g/ml lidocaine (Jetokaine®) were administered to all patients. Superior and temporal corneal incisions were made. Sodium hyaluronate (3.0%) and sodium chondroitin sulfate (4.0%) were used to maintain the anterior chamber and protect the corneal endothelium. Capsulorhexis of 5.0-5.5 mm diameter was performed with Utrata forceps. In cases without visible fundus reflex due to severe cataract, capsulorhexis was completed after staining the anterior lens capsule with 0.1% trypan blue. The quick-chop phacoemulsification technique was used with balanced salt solution hydrodissection and hydrodelineation. The IOL was implanted through the temporal incision, which was not used for phacoemulsification, after it was widened to accommodate the lens. The incision was closed by stromal hydration. Eyes with Eyecryl micro 262 IOLs implanted through 1.6 mm corneal incisions comprised group 1; eyes with Optiflex MO/F-13 IOLs implanted through corneal incisions widened to 2.0 mm comprised group 2. Group 3 consisted of eyes with 2.4 mm corneal incisions and Acriva UD 613, Zaraccom Focus Force Basic IOLs, while in group 4 the corneal incisions were widened to 2.8 mm for Alcon SN60AT IOLs. Postoperatively all patients were administered ofloxacin (Exocin 0.3%) and 1% prednisolone acetate (Pred-Forte) five times a day. In both clinics keratometry (K) values were measured preoperatively and 1 month postoperatively using an autorefractometer/keratometer (Nidek ARK-510A), followed by evaluation of SIA based on the method developed by Holladay et al.^18^

Statistical analyses were performed using Statistical Package for the Social Sciences version 16.0. One-way ANOVA was used to compare the four groups. Post hoc analysis was used for pairwise comparisons.

## RESULTS

**Figure 1:**
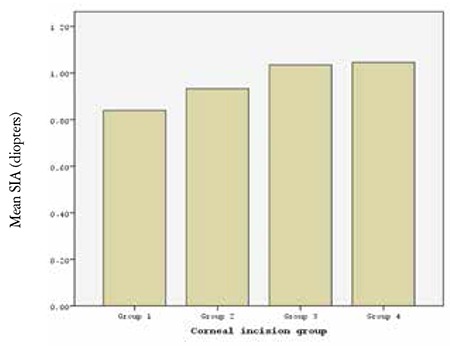
Comparison of the surgically induced astigmatic changes in the study groups. SIA: Surgically induced astigmatism

Table 1 presents patient age, sex, nuclear hardness grades, and pre- and postoperative mean K values of 683 eyes in 4 groups based on corneal incision size during biaxial microincision phacoemulsification surgery. SIA was 0.83±0.4 diopter (D) in group 1, 0.93±0.5 D in group 2, 1.03±0.6 D in group 3 and 1.04±0.7 D in group 4. Comparison of the four groups revealed a statistically significant difference (p=0.05). In pairwise group comparisons, group 1 was significantly different than groups 3 and 4 (Figure 1, Table 2).

Each group was divided into tertiles according to preoperative K values. In group 1, the mean SIA was 0.93±0.4 D in the highest K tertile versus 0.79±0.4 D in the lower two tertiles (p=0.216). These values were 1.00±0.7 D and 0.89±0.5 D in group 2 (p=0.251), 1.08±0.7 D and 1.00±0.5 D in group 3 (p=0.436), and 0.97±0.6 D and 1.07±0.8 D in group 4 (p=0.384).

In each group, mean SIA values in eyes with grade I-II nuclear hardness and grade III-IV nuclear hardness were compared. In group 1, SIA was 0.75±0.4 D in grade I-II versus 0.92±0.5 D in grade III-IV (p=0.129). These values were 0.83±0.5 D and 1.01±0.5 D in group 2 (p=0.032), 0.96±0.6 D and 1.07±0.7 D in group 3 (p=0.265), and 1.00±0.7 D and 1.08±0.8 D in group 4 (p=0.470).

## DISCUSSION

With the steady reduction of corneal incision sizes and the realization that astigmatism may be corrected during the procedure, cataract surgery is increasingly accepted as a refractive surgery as well as a sight-restoring operation. Accordingly, patients’ expectations of having excellent uncorrected near and distance vision postoperatively are increasing. However, SIA impacts both best visual acuity and visual rehabilitation time and continues to be a major concern for surgeons, who have attempted to minimize the problem by adjusting the location and size of the corneal incisions.

In the B-MICS procedure, the number of corneal incisions was reduced from the traditional three to two, and their size was reduced from the 2.2-3.2 mm range to 1.5 mm or less. B-MICS provides better anterior chamber stabilization and faster healing. The procedure also creates less damage to tissues adjacent to the cornea and substantially reduces SIA and corneal aberrations.^19,20^ Despite the studies that have reported successful outcomes with IOLs that can be implanted through small incisions, these lenses may not be practical due to their excessive cost or lack of multifocal or toric versions. Therefore, corneal incision enlargement may be necessary after B-MICS, which poses a limitation to this method.^8,17^

In this study we evaluated how enlarging the corneal incision not used for phacoemulsification in order to implant the IOL after benefiting from the advantages of B-MICS affects SIA. Masket and Tennen^21^ observed corneal curvature stabilization in postoperative week 2 in patients with corneal incisions ≤3 mm. Using this study as a reference, in the current study we used K values from postoperative 1 month to calculate SIA.

Wang et al.^22^ evaluated astigmatism resulting from microincision and small incision cataract surgery and found astigmatism values of 0.5±0.5 D for 2.2 mm incisions, 0.6±0.5 D for 2.6 mm incisions and 0.9±0.9 D for 3.0 mm incisions. Although the difference between 2.2 and 2.6 mm was insignificant, there was a significant difference between 2.2 mm and 3.0 mm. In the same study, the authors concluded that 2.2 mm and 2.6 mm incisions lead to lower SIA and earlier refractive stabilization, thus allowing for more rapid visual rehabilitation. In a study by Can et al.^23^ comparing coaxial, micro-coaxial and biaxial MICS, based on the final incision length after IOL implantation, 2.83 mm incision caused astigmatism of 0.46 D, 2.26 mm caused 0.24 D and 1.89 mm caused 0.13 D astigmastism, which was a statistically significant difference. Kaya et al.24 found that in 25 cases, enlarging the corneal incision from 1.5 mm to 2.0 mm for IOL implantation after phacoemulsification resulted in astigmatism of 0.44±0.36 D. In the present study, although we did not detect a significant difference between the amount of induced astigmatism in eyes that underwent 1.6 mm B-MICS and eyes with incisions enlarged to 2.0 mm, there were significant differences between eyes with 1.6 mm incisions and those with 2.4 mm and 2.8 mm incisions.

Although there are studies reporting no difference between B-MICS, MICS and standard phacoemulsification procedures in terms of effective phaco time, total phaco time and total surgery time, some studies have demonstrated shorter effective phaco time and longer phaco time in B-MICS.^25,26,27,28^ Effective phaco time is shorter and total phaco time is longer with hard cataracts, but no significant differences emerged in our analysis of the association between SIA and cataract severity. Furthermore, we found no significant difference between SIA in eyes with the highest K values versus eyes with lower K values.

## CONCLUSION

In summary, enlarging corneal incisions up to 2.00 mm to implant the IOL after B-MICS does not significantly increase SIA. However, as incision size increases to 2.8 mm, the difference in SIA becomes significant. Regardless of the need to enlarge small incisions to accommodate IOLs during implantation, having two small incisions in B-MICS provides better control of the anterior chamber and therefore can reduce intraoperative complications. Despite an SIA difference of up to 0.21 D, B-MICS is preferrable due to its faster postoperative visual rehabilitation. We believe that the development and widespread availability of IOLs implantable through microincisions will increase the value of B-MICS in cataract surgery.

## Ethics

Ethics Committee Approval: Retrospective study, Informed Consent: Retrospective study.

Peer-review: Externally and internally peer-reviewed.

## Figures and Tables

**Table 1:**
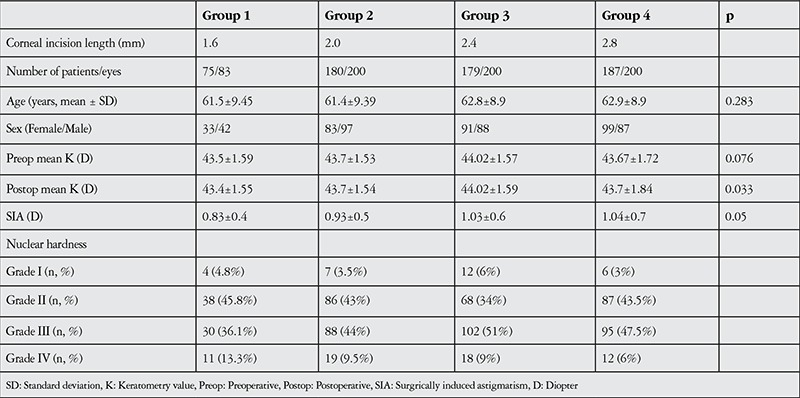
Demographic and clinical characteristics of the patients by group

**Table 2:**
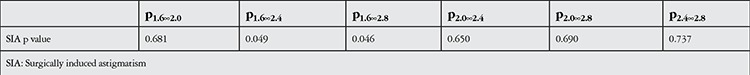
Pairwise comparisons of surgically induced astigmatic changes in groups with different corneal incision lengths (mm)
